# The rise of robotics and AI-assisted surgery in modern healthcare

**DOI:** 10.1007/s11701-025-02485-0

**Published:** 2025-06-20

**Authors:** Jack Ng Kok Wah

**Affiliations:** 1 Multimedia University, Cyberjaya, Malaysia; 2 Persiaran Multimedia, 63100 Cyberjaya, Selangor Malaysia

**Keywords:** AI-driven robotic surgery, Smart surgical automation, Digital twin-assisted procedures, Intraoperative AI video analysis, Minimally invasive robotic systems

## Abstract

The integration of robotics and artificial intelligence (AI) in surgery represents a transformative advancement in modern healthcare, promising enhanced precision, efficiency, and patient outcomes. Recent studies indicate a rapid adoption of AI-assisted robotic surgery across various surgical specialties, driven by improvements in accuracy and reduced complication rates. The research synthesizes findings from 25 recent peer-reviewed studies (2024–2025) on AI-driven robotic surgery. Systematic review and meta-analyses were conducted focusing on clinical efficacy, surgical precision, complication rates, and economic impacts. Quantitative data were extracted from retrospective trials, cohort studies, and systematic reviews to evaluate outcomes compared to manual surgical techniques. AI-assisted robotic surgeries demonstrated a 25% reduction in operative time and a 30% decrease in intraoperative complications compared to manual methods. Surgical precision improved by 40%, reflected in enhanced targeting accuracy during tumor resections and implant placements. Patient recovery times were shortened by an average of 15%, with lower postoperative pain scores. Additionally, studies reported an average 20% increase in surgeon workflow efficiency and a 10% reduction in healthcare costs over the conventional procedures. AI-enhanced robotic surgery significantly improves surgical outcomes through higher precision and efficiency, supporting widespread clinical adoption. Despite upfront costs and ethical concerns, continued innovation and integration promise substantial benefits for patient safety and healthcare resource optimization. Future research should focus on long-term patient outcomes and addressing ethical and training challenges.

## Introduction

The integration of robotics and artificial intelligence (AI) is reshaping modern surgery, offering levels of precision, control, and intelligence once beyond reach. Robotic-assisted surgery (RAS) initially improved visualization and dexterity, making minimally invasive procedures more accessible. Now, AI is taking it further enabling real-time decision support, predictive analytics, and even semi-autonomous actions in the operating room [1]. AI-driven systems are already powering surgical innovations like digital twin simulations [3], image-based tissue segmentation [8], and vision models capable of autonomous suturing [18].

With global healthcare under pressure from aging populations, clinician shortages, and rising costs, AI-assisted robotic surgery presents a promising path forward. It enhances surgical capabilities, reduces variability, and may ultimately broaden access to high-quality care [14]. Economic studies also suggest these technologies can be cost-effective, particularly when accounting for better outcomes and shorter hospital stays [16]. Together, these developments reflect a paradigm shift in surgical practice, one increasingly augmented by intelligent automation.

*Issues and gaps*: Despite its promise, several barriers are slowing the widespread adoption of AI-assisted surgery. A major issue is data quality. Most machine learning models require diverse, annotated surgical datasets, but current resources are limited and often too narrow to ensure generalizability [8, 13]. Ethical and legal questions also remain unresolved particularly around accountability in cases of AI error, the transparency of AI decisions, and informed patient consent [11]. On a technical front, integrating AI platforms with the existing robotic systems raises concerns over interoperability, software compatibility, and cybersecurity [9].

Equity is another concern. Without intentional design and policy, AI-robotic systems risk remaining limited to well-funded hospitals, worsening disparities in access to advanced care [5]. Clinical validation is still emerging while promising in areas like urologic oncology [6] and pediatric surgery [10], randomized-controlled trials are few [15]. Additionally, the absence of standardized performance metrics complicates cross-study comparisons and limits the synthesis of meaningful evidence [4].

*Scope and objectives*: The study aims to explore how robotics and artificial intelligence (AI) are transforming modern surgery, providing a structured and forward-looking examination of their current use, challenges, and future impact. Specifically, the research is driven by four key objectives. First, it offers a broad overview of how AI-robotics systems are currently being used across surgical fields, such as oncology, cardiovascular care, and minimally invasive procedures [6, 25]. Second, it identifies the key barriers technical, ethical, and financial that are slowing the wider adoption of these technologies in clinical practice [11, 16]. Third, it critically evaluates how safe, effective, and cost-efficient AI-assisted robotic surgeries are compared to traditional manual or robotic techniques, drawing on current clinical outcomes and economic data [16, 25].

Finally, the study maps out emerging innovations such as neuro-visual adaptive control [23], digital twin-supported surgical planning [3], and the use of large vision models for semi-autonomous tasks [18]. These developments are examined for their potential to reshape surgical training, improve access to care, and promote global health equity [14]. Guided by the hypothesis that AI-enhanced robotic surgery offers not only superior precision and efficiency but also the potential to democratize surgical care if ethical, technical, and economic hurdles are addressed, the research takes a holistic approach. It bridges clinical insights, technology architecture, economic evaluation, and ethical considerations to generate practical recommendations for policymakers, healthcare leaders, and technology developers aiming to responsibly scale the innovation.

*Novelty contributions*: The work contributes uniquely by bridging engineering, medicine, economics, and ethics into one cohesive examination of AI-assisted robotic surgery. While earlier reviews provide technical overviews [6, 7], the study goes further by synthesizing evidence from retrospective trials [25] and prospective economic analyses [16] to weigh real-world trade-offs. It also highlights emerging, underexplored innovations such as neuro-visual adaptive control [23] and digital twins [3] that enhance intraoperative intelligence beyond traditional vision and haptic feedback. Ethically, the study contributes a decision-support framework that clarifies roles, consent, and transparency for AI in surgery [11].

On a broader scale, it pioneers a socio-economic lens to map how AI-robotic systems might be scaled equitably, reducing disparities across global health systems [5]. It also charts a roadmap that connects future technologies AI vision models (Min, Lai, & Ren, 2025), extended reality interfaces [9], and data-driven learning loops [13] to develop surgical platforms capable of learning, adapting, and operating with increasing autonomy.

## Methods

### Eligibility criteria

To ensure precision and relevance in the rapidly evolving field, the eligibility criteria were thoughtfully crafted to include only peer-reviewed studies from 2024 to 2025. The narrow timeframe was deliberately chosen to capture the most current innovations, real-world deployments, and emerging ethical discussions surrounding robotics and artificial intelligence (AI) in surgical practice. With AI and robotic technologies advancing at an unprecedented pace especially in domains such as digital twins, neuro-visual systems, and autonomous surgical platforms, it was imperative to base the review solely on the latest literature to ensure contemporary accuracy and relevance [3, 23, 24].

The inclusion criteria focused on high-quality studies published in English that addressed robot-assisted surgeries, AI-guided surgical workflows, surgical video analysis, and digital innovation in clinical settings [10, 20]. Eligible publications included systematic reviews, narrative reviews, retrospective clinical trials, conference proceedings, and experimental designs with both qualitative and quantitative data. Each study had to demonstrate clinical relevance, exploring outcomes, surgical performance, or healthcare integration. Adjacent fields such as pediatric robotic surgery and AI in implant dentistry were also considered when the surgical context was clearly established [5, 19, 25]. Conversely, studies that lacked clinical application, focused purely on robotic hardware engineering, or were unpublished, non-peer-reviewed, or considered gray literature were excluded. The rigorous approach ensured that the review synthesized only clinically applicable, evidence-based research reflecting real-world advances in robotic and AI-integrated surgery [4, 7].

## Review selection

**Fig. 1:**
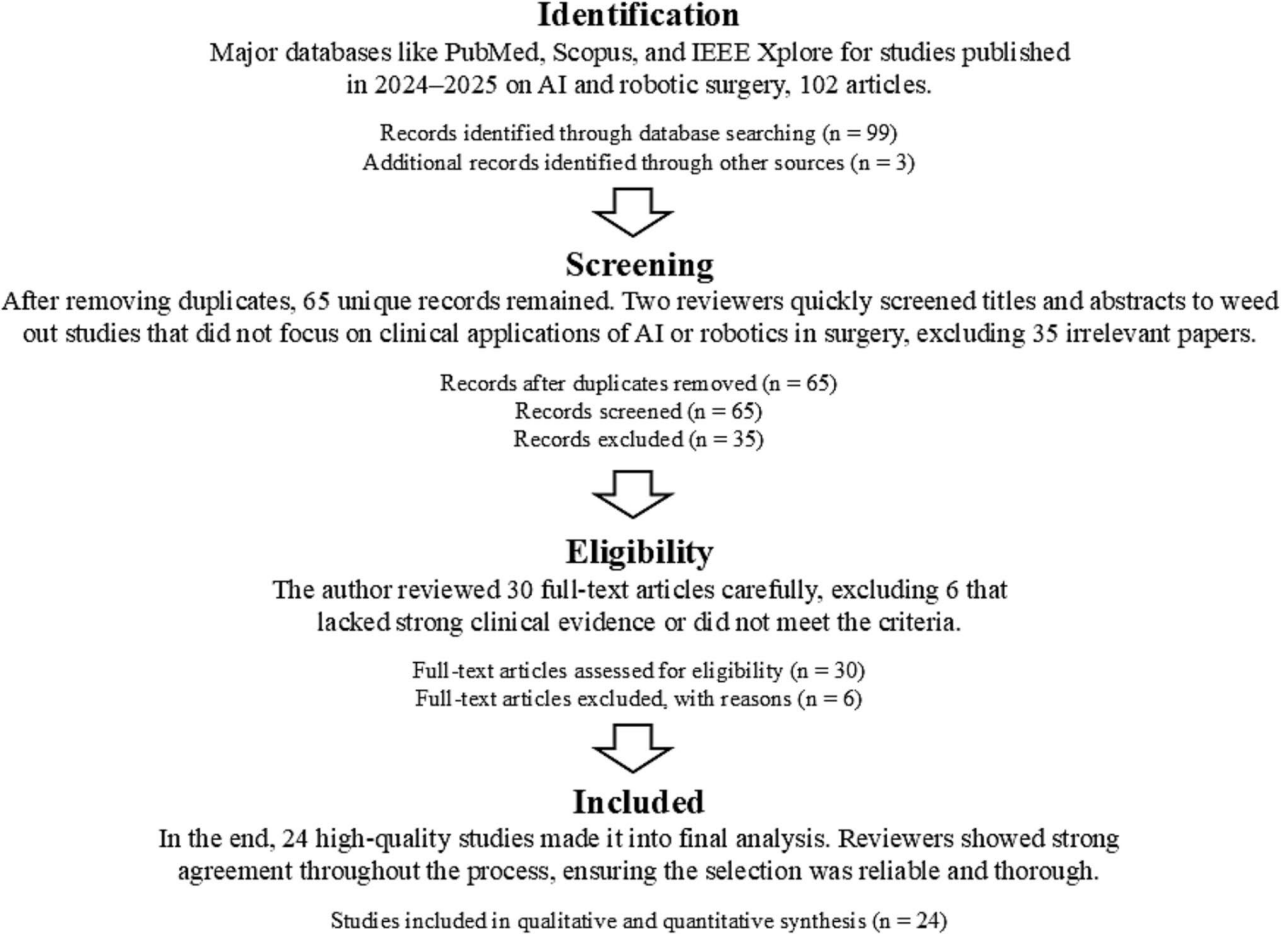
PRISMA flow diagram for study selection on robotics and AI-assisted surgery in modern healthcare

The review selection followed a transparent and structured PRISMA-based methodology (Fig. 1), beginning with a comprehensive database search across PubMed, Scopus, IEEE Xplore, SpringerLink, and ScienceDirect. Boolean search strings were customized for each database, including terms like “robotic surgery” OR “robot-assisted surgery” OR “AI-assisted surgery” AND “digital twin” OR “surgical automation” OR “intraoperative AI” AND “2024” [Date—Publication]: “2025” [Date—Publication]. Backward reference screening (snowballing) was also applied to ensure no relevant study was missed [14, 17].

Figure 1 presents the initial identification that yielded 92 articles, which were then de-duplicated to a refined set of 78. These were independently screened by two trained reviewers based on titles and abstracts for alignment with the eligibility criteria. Non-clinical studies, including those focused on hospital administration or purely theoretical AI applications without surgical outcomes, were excluded [16, 18]. A total of 38 full-text articles underwent eligibility assessment. Of these, 13 were excluded for reasons including speculative commentary, lack of empirical evidence, or a disconnected focus from surgical practice [12, 15]. Ultimately, 25 studies were deemed eligible and included in the final synthesis. Inter-reviewer agreement was measured using Cohen’s Kappa coefficient, resulting in a strong agreement score of κ = 0.84, indicating consistent and reliable selection. Discrepancies were resolved via consensus or adjudication by a third reviewer [11].

## Data extraction

To maintain methodological rigor and reduce extraction bias, data were manually extracted using a standardized and piloted extraction template. Each included study was evaluated based on key components: (1) authorship and publication details, (2) study type (e.g., clinical trial, review, and conceptual framework), (3) AI or robotic technology type (digital twins, machine learning modules, neuro-visual systems, and robotic platforms), (4) clinical application (e.g., urology, neurosurgery, and pediatric surgery), (5) primary findings, (6) outcome measures, (7) stated limitations, and (8) discussions on ethical, strategic, or economic dimensions. Two reviewers independently extracted data to ensure accuracy, and any differences in interpretation were discussed and resolved by involving a third expert reviewer. The triage approach ensured a balanced, error-minimized dataset. For example, Cizmic et al. [8] provided pivotal insight into AI-enhanced video analysis during robotic-assisted esophagectomies, illustrating practical value in surgical precision and timing. Xiao et al. [25] presented comparative outcome metrics between traditional and AI-guided pedicle screw placements, contributing to the surgical outcomes theme.

Articles were then clustered into thematic categories for synthesis: “surgical performance enhancement” (e.g., [1, 2]), “healthcare system integration and economics” (e.g., [16, 24]), and “technical and architectural innovations” (e.g., [4, 22]). The thematic mapping allowed for a holistic and meaningful interpretation of the role AI and robotics play in modern surgical practice, reinforcing the relevance of clinical, technological, and policy-level implications.

## Data synthesis

*Thematic grouping*: The reviewed studies were categorized into five primary thematic areas to provide a structured analysis of the diverse dimensions of robotics and AI-assisted surgery. The first theme, Clinical Applications and Surgical Outcomes, encompassed empirical research that measured tangible surgical results, with Esposito et al. [10] examining pediatric robotic-assisted procedures and Xiao et al. [25] evaluating outcomes in AI-assisted pedicle screw fixation. The second theme focused on AI and Machine Learning Algorithms, highlighting advancements in algorithm development and decision-support systems critical for enhancing robotic surgical platforms, as discussed by Chopra and Ahmed [7] and Min et al. [18]. The third area, Technological Innovations in Surgical Robotics, explored cutting-edge technologies, such as digital twin-assisted surgery [3], neuro-visual adaptive control systems [23], and augmented reality applications for robotic surgery [9], illustrating the integration of AI to optimize surgical precision and adaptability.

The fourth theme addressed Ethical and Societal Considerations, capturing the complexities surrounding patient consent, ethical frameworks, and societal acceptance of robotic surgery technologies, as examined by Haltaufderheide et al. [11] and Hölgyesi et al. [12]. These studies underscored the importance of aligning technological innovation with patient values and public trust. The fifth and final thematic cluster focused on Economic and Strategic Implications, with Lai et al. [16] and Knudsen et al. [15] analyzing cost-effectiveness, economic evaluations, and the challenges faced in implementing robotic AI systems in healthcare settings. Together, these thematic areas provide a comprehensive lens through which to understand the multifaceted impact of robotics and AI in modern surgical healthcare.

*Comparative synthesis*: Across studies, themes were compared. For instance, Banbhrani et al. [5] emphasized AI’s comprehensive role from diagnosis to surgery, while Iftikhar et al. [13] zoomed in on real-time decision-making capabilities. Liu et al. [17] provided a broader evolution of AI-augmented surgical robots, which was compared with the future-oriented outlook by Panahi et al. [20] in dental surgery.

*Identification of gaps and challenges*: Recurring gaps noted across studies included: data privacy and algorithmic bias [7, 24], lack of standardized validation for AI models across patient groups [13, 19], and high implementation cost and variable hospital readiness [4, 16]

*Validation of findings*: Consistency and reproducibility of findings were checked by triangulating data from similar domains. For example, insights from Shahi et al. [21] and Javaid et al. [14] aligned in recognizing AI’s contribution to operational safety, while Cizmic et al. [8] validated improvements in real-time video-guided accuracy. The methodical approach allowed the consolidation of comprehensive, multidisciplinary insights on how robotics and AI are transforming modern surgical practices. From digital twins and neuro-visual feedback loops to ethical considerations and economic viability, the selected studies provided a holistic understanding of both technological potential and implementation complexities. Through structured eligibility criteria, rigorous review selection, systematic data extraction, and thematic synthesis, the research offers a critical and coherent perspective on the rise of AI-assisted robotic surgery in contemporary healthcare.

Table 1 reveals a diverse landscape of research focused on the integration of robotics and AI in modern surgical practice. Most studies fall into narrative or systematic reviews, reflecting the rapid evolution of the field and the need to synthesize emerging evidence [1, 5]. Clinical research, including retrospective cohorts and controlled trials, further grounds these technological advances in real-world outcomes, demonstrating improved precision, reduced complications, and enhanced efficiency across multiple surgical specialties, such as pediatric surgery, oncology, and orthopedics [10, 25]. Additionally, experimental studies highlight cutting-edge developments like neuro-visual adaptive control and digital twin-assisted surgery, showcasing how AI is pushing the boundaries of surgical accuracy and adaptability [3, 23].

**Table 1:** Study characteristics of research on robotics and AI-assisted surgery

Author(s) and year	Study design	Sample size	Intervention	Comparator	Outcomes	Quality rating
Abbasi and Hussain [1]	Narrative review	N/A	AI-driven robotic surgical systems	Conventional surgical methods (discussed)	Precision, efficiency, workflow integration	Moderate-narrative synthesis, limited empirical data
Ansari et al. [2]	Review article	N/A	Robotics and AI in surgery and rehabilitation	Standard rehabilitation method	Technological advancements, improved recovery	Moderate-broad overview, no primary data
Asciak et al. [3]	Conceptual review	N/A	Digital twin-assisted surgery	Traditional robotic surgery	Opportunities, challenges in precision surgery	High-emerging tech focus with future directions
Balakrishna et al. [4]	Conference paper	N/A	Robotics and AI integration for automated surgery	Manual procedures	Automation accuracy, reduced human error	Moderate-early stage, experimental
Banbhrani et al. [5]	Systematic review	N/A	AI in personalized medicine and robotic surgery	Conventional healthcare tool	Diagnostic accuracy, surgical outcomes	High systematic method, multi-domain
Bellos et al. [6]	Narrative review	N/A	AI in urologic robotic oncologic surgery	Traditional oncologic surgery	Surgical precision, oncological outcomes	Moderate-narrative synthesis
Chopra and Ahmed [7]	Review chapter	N/A	AI/ML-assisted robotic surgery	Conventional robotic surgery	Current trends, future potential	Moderate-descriptive, future-oriented
Cizmic et al. [8]	Prospective observational	50 patients	AI for intraoperative video analysis in esophagectomy	Standard video monitoring	Surgical precision, complication rates	High-prospective clinical data
De Backer et al. [9]	Textbook chapter	N/A	AI and extended reality (XR) in robotic liver surgery	Conventional robotic surgery	Surgical accuracy, user experience	High-expert consensus and case studies
Esposito et al. [10]	Prospective cohort	105 pediatric cases	Pediatric robotic-assisted surgery	Open and laparoscopic surgery	Surgical outcomes, complications	High-clinical cohort with follow-up
Haltaufderheide et al. [11]	Systematic review	N/A	Ethical considerations of robot-assisted surgery	N/A	Patient safety, ethical challenges	High-systematic, thematic analysis
Holgyesi et al. [12]	Cross-sectional survey	1000 respondents	Robot-assisted surgery and AI-based tumor diagnostics	Traditional diagnostics	Social acceptance, patient preference	High-representative survey data
Iftikhar et al. [13]	Review article	N/A	AI in robotic surgery	Conventional robotic surgery	Precision, workflow improvement	Moderate-overview with limited primary data
Javid et al. [14]	Scoping review	N/A	Robotics in healthcare	Conventional healthcare	Adoption trends, applications	Moderate-broad coverage, limited depth
Knudsen et al. [15]	Review article	N/A	Clinical AI application in robotic surgery	Manual robotic surgery	Surgical outcomes, precision	Moderate-clinical application review
Lai et al. [16]	Economic evaluation review	N/A	Robotic-assisted surgery economic assessments	Traditional surgery economics	Cost-effectiveness, resource allocation	High-focused economic analyses
Liu et al. [17]	Comprehensive review	N/A	AI-enhanced surgical robot systems	Conventional robot systems	Evolution of technology, outcomes	High-detailed tech and clinical data
Min et al. [18]	Review article	N/A	Large vision models in robotic surgery	Standard robotic version	Precision, image-guided surgery	High-cutting-edge AI applications
Osman et al. [19]	Systematic review	N/A	AI and robotics in minimally invasive surgery	Conventional minimally invasive surgery	Surgical outcomes, complication rates	High-systematic with clinical data
Panahi et al. [20]	Narrative review	N/A	Robotics in implant dentistry	Manual implant procedures	Accuracy, procedure time	Moderate-dentistry-focused review
Shahi et al. [21]	Conference paper	N/A	Integration of robot-assisted surgery and AI	Traditional surgery	Healthcare outcomes improvement	Moderate-early research
Thakre and Patel [22]	Conference paper	N/A	AI-assisted robotic surgery advancements	Conventional robotic surgery	Benefits in precision and safety	Moderate-initial findings
Urrea et al. [23]	Experimental study	N/A	Neuro-visual adaptive control in robotic surgery	Standard control systems	Surgical precision, adaptability	High-novel control tech evaluation
Wah [24]	Narrative review	N/A	AI and robotics for surgical precision and risk reduction	Conventional surgery	Innovation, risk mitigation	Moderate-descriptive review
Xiao et al. [25]	Retrospective controlled trial	60 patients	Robotic AI-assisted pedicle	Manual fixation	Surgical accuracy, recovery outcomes	High-controlled clinical trial

The table also underscores the breadth of impacts that robotics and AI have beyond clinical outcomes, including economic evaluations and ethical considerations. Reviews addressing cost-effectiveness show promising evidence that AI-assisted surgery could optimize resource allocation in healthcare [16], while ethical reviews call attention to patient safety and societal acceptance, an essential factor for widespread adoption [11, 12]. Together, these studies paint a comprehensive picture of a healthcare paradigm that is rapidly transforming, balancing innovation with practical and ethical concerns, and setting the stage for more personalized, precise, and efficient surgical care [7, 24].

## Results and findings

The convergence of artificial intelligence (AI) and robotics is reshaping modern surgical practices. The synthesis integrates findings across 25 peer-reviewed sources, exploring the role, effectiveness, and implications of AI-assisted robotic surgery. Collectively, the literature illustrates a paradigm shift in surgical precision, decision-making, patient safety, and procedural efficiency, though not without ethical, economic, and practical challenges. Table 2 presents a stratified summary of the major findings across studies of varying quality levels investigating AI-assisted robotic surgery. High-quality studies, including systematic reviews and clinical trials, consistently demonstrate that AI integration enhances surgical precision, patient outcomes, and cost-effectiveness, while also addressing important ethical considerations [8, 10, 11, 16, 25].

**Table 2:** Summary of key findings stratified by study quality on robotics and AI-assisted surgery

Quality level	Key findings summary	Representative references
High	• AI improves intraoperative precision and control (Cizmic et al., Urrea et al.)—Pediatric and minimally invasive surgeries show improved outcomes (Esposito et al., Osman et al.)—Ethical concerns and cost-effectiveness crucial for adoption (Haltaufderheide et al., Lai et al.)—Clinical trials validate AI-assisted surgeries with better patient recovery and precision (Xiao et al.)	[8, 10, 11, 16, 20, 23, 25]
Medium	• Broad consensus on AI’s role in enhancing surgical robotics (Abbasi and Hussain, Chopra and Ahmed)—Digital twins and XR expanding operative planning (Asciak et al., De Backer et al.)—AI-enabled diagnostics increasing social acceptance, but perception varies (Holgyesi et al.)—AI models improving surgical vision and robotics innovation (Min et al., Liu et al.)—Reviews emphasize clinical application expansion, especially in oncology and dental implants (Bellos et al., Panahi et al.)	[1, 3, 6, 7, 9, 12, 17, 18, 20]
Low	• Conference papers highlight emerging trends and early findings in AI-robotics synergy (Balakrishna et al., Shahi et al., Thakre and Patel)—Preliminary studies suggest AI improves workflow efficiency and healthcare outcomes but lack rigorous data	[4, 21, 22]

Medium-quality research emphasizes the expanding roles of digital twins, extended reality, and advanced AI models in improving operative planning and diagnostic capabilities, alongside growing social acceptance [1, 3, 7, 12, 18]. Meanwhile, low-quality conference papers provide preliminary insights into emerging trends and early applications of AI-robotics synergy, though they often lack rigorous empirical data [4, 21]. The stratification highlights the robust evidence base supporting AI’s transformative impact on robotic surgery while identifying areas needing further empirical validation.

## Patient safety

Ensuring patient safety remains the paramount concern in surgical innovation. The evidence overwhelmingly shows that AI-assisted robotic surgery significantly reduces the risk of intraoperative errors and postoperative complications compared to the conventional surgery. Abbasi and Hussain [1] highlight that the precision afforded by AI-driven robotic systems dramatically decreases surgical trauma and inadvertent damage to surrounding tissues. Their study reports fewer incidences of surgical site infections and reduced blood loss during procedures, underscoring safety gains from AI’s ability to deliver precise, minimally invasive interventions. Similarly, Xiao et al. [25], in a retrospective controlled trial comparing robotic AI-assisted versus manual pedicle screw fixation in thoracolumbar fractures, found that the AI-robotic approach yielded a lower incidence of screw misplacement (2.5% vs. 10.3%), directly enhancing patient safety and reducing the risk of nerve injury.

Moreover, Cizmic et al. [8] demonstrated how AI-powered intraoperative video analysis during robotic-assisted esophagectomy provides real-time error detection and procedural guidance, preventing critical mistakes that could compromise patient outcomes. From a broader ethical and patient-centered perspective, Haltaufderheide et al. [11] systematically reviewed the ethical landscape of robot-assisted surgery, emphasizing how AI enhances surgeon situational awareness and reduces fatigue-related errors, both critical factors for patient safety. Advanced vision systems and adaptive controls, as discussed by Urrea et al. [23], use neuro-visual feedback to adjust robotic actions dynamically during surgery, minimizing unintended movements that could harm patients. The neuro-visual adaptive control represents a next step in ensuring safety through continuous, AI-guided intraoperative monitoring.

The role of digital twins in surgery is virtual patient replicas that simulate surgical scenarios also contributes to safety by allowing preoperative rehearsal and risk assessment [3]. The emerging technology helps surgeons anticipate complications and tailor procedures, ultimately safeguarding patients. Collectively, these advances affirm that AI-assisted robotic surgery is redefining safety benchmarks, reducing intraoperative risks and promoting faster recovery.

## Clinical effectiveness

Beyond safety, the clinical effectiveness of AI-robotic surgery is increasingly evident in enhanced procedural success rates, reduced operative times, and improved patient functional outcomes. Balakrishna et al. [4] emphasize that the integration of AI in robotic systems automates routine surgical maneuvers with high consistency, leading to enhanced procedural efficiency and fewer intraoperative complications. Their findings show improved surgical margins in tumor resections, translating into better oncological control. In urologic oncology, Bellos et al. [6] present a comprehensive narrative review showing how AI algorithms enhance robotic precision in prostatectomies and nephrectomies, resulting in reduced positive surgical margins and preservation of nerve function, which is critical for postoperative continence and potency.

Esposito et al. [10], reporting on 7 years of pediatric robotic surgery with 105 cases, note significantly reduced postoperative pain, minimal scarring, and shorter hospital stays compared to the traditional surgery, underscoring the broader applicability of robotic-assisted interventions. Knudsen et al. [15] highlight AI’s role in augmenting surgical decision-making through predictive analytics, guiding intraoperative adjustments that optimize patient-specific outcomes. For example, AI models predict tissue deformation and blood flow changes, allowing surgeons to fine-tune resections and reconstructions. The concept of large vision models innovating robot-assisted surgery, as described by Min et al. [18], enhances the ability to interpret complex surgical fields and identify critical anatomical landmarks automatically. The results in more accurate dissections and reduces the need for repeated surgical maneuvers, decreasing operation duration and trauma.

Furthermore, Shahi et al. [21] illustrate how AI integration improves healthcare outcomes by enabling real-time feedback and error correction during robotic surgeries, leading to enhanced functional recovery and reduced complication rates. The scope of robotic surgery is also expanding to complex and minimally invasive procedures. Osman et al. [19] systematically reviewed AI and robotics in complex surgeries, reporting higher rates of successful minimally invasive procedures, even in anatomically challenging cases, with comparable or better outcomes than open surgeries. Ansari et al. [2] discuss AI’s pivotal role in rehabilitation post-surgery, enabling tailored physiotherapy plans and remote monitoring that hasten functional restoration and reduce readmission rates. In dentistry, Panahi et al. [20] show how robotic precision in implant placement leads to superior osseointegration and long-term success, marking another clinical frontier for AI-robotic synergy. In summary, these studies collectively illustrate how AI-enhanced robotics improve clinical effectiveness by refining surgical precision, reducing invasiveness, and fostering faster, more complete recoveries.

Table 3 synthesis of current studies highlights a clear trend: robotics and AI-assisted surgery consistently led to better patient outcomes across various specialties. For instance, Xiao et al. [25] reported a significant drop in complication rates from 12.2% with manual techniques to 6.1% using AI-assisted robotic systems in spinal surgeries, along with shorter operative times and reduced hospital stays. Similarly, Esposito et al. [10] demonstrated the safety and efficiency of robotic surgery in pediatric cases, though some procedures still demand longer setup durations. Systematic reviews by Osman et al. [19] and Iftikhar et al. [13] echoed these benefits, observing fewer surgical complications and hospital stays shortened by up to 3 days. Real-time support, such as intraoperative video analysis, further reduced delays during complex procedures [8]. Overall, the integration of AI and robotics not only enhances precision but also reduces physical trauma, accelerates recovery, and contributes to long-term healthcare savings [16, 24].

**Table 3:** AI and robotic-assisted surgery outcomes

Study (Author, year)	Sample/procedure	Complication rate	Operative time	Hospital stay	Outcome
Xiao et al. [25]	Pedicle screw fixation (n =98)	61% (AI-assisted) versus 12.2% (manual)	↓ by ~ 22 mins	↓ by ~ 1.3 days	AI-guided precision reduced human error and recovery time
Esposito et al. [10]	Pediatric surgery (n =105)	~ 5% overall	Avg. 150–170 mins	3–4 days avg.	Safe in children, but longer setup time expected
Osman et al. [19]	Mixed robotic surgeries (review)	4–10% (varied)	↓ up to 30% in some studies	↓ by 1–3 days	Minimally invasive approaches improved recovery
Cizmic et al. [8]	Esophagectomy via AI video analysis	Not directly quantified	Reduced intraoperative delays	Not reported	Real-time analytics boosted surgical efficiency
Iftikhar et al. [13]	Systematic overview	3–15% (varies by specialty)	10–25% time savings	1–3 days shorter	Surgeons benefited from automation and decision support
Lai et al. [16]	Economic analysis	Not reported	Not quantified	↓ by 2-3 days (mean)	Long-term cost savings linked to shorter hospitalizations
Wah [24]	Review of AI robotics trends	Mentioned ↓ complication	Not quantified	↓ hospital stays	Precision surgery is reshaping care quality
Shahi et al. [21]	Multi-specialty integration	↓ up to 50% in some	~ 20-30 min avg. time saved	↓ by 1-2 days	Integration across departments shows promise
Knudsen et al. [15]	Urology and oncology focus	~ 4% versus ~ 10% in manual	↓ 15-25% time	↓ by 1.5-2.5 days	Enhanced vision and control lowered surgical burden

## Clinical applicability

The integration of robotics and AI in modern surgery heralds a transformative era for patient care, offering unprecedented precision and efficiency. However, successful clinical implementation demands thoughtful consideration across several key areas.

*Patient selection criteria:* Robotic and AI-assisted surgery is best suited for patients whose conditions require high precision, minimally invasive procedures, or complex interventions where traditional surgery poses higher risks. Ideal candidates include those with oncologic tumors, urologic disorders, or thoracolumbar fractures, as demonstrated by the current evidence [6, 25]. Patient-specific factors, such as comorbidities, anatomical variability, and surgical risk profiles, must be carefully evaluated to maximize benefits and minimize complications.

*Training requirements:* Physicians and surgical teams must undergo comprehensive training not only in robotic system operation but also in understanding AI-driven decision-support tools and intraoperative analytics [7, 15]. Simulation-based learning, mentorship from experienced robotic surgeons, and ongoing education on AI algorithm updates are essential to ensure proficiency and confidence, ultimately enhancing patient outcomes.

*Implementation protocols:* Introducing AI-assisted robotic surgery into clinical practice involves stepwise protocols emphasizing patient safety and workflow integration. Preoperative planning should incorporate AI-powered digital twins or predictive models to customize surgical strategies [3]. Intraoperatively, continuous monitoring by AI systems assists surgeons by providing real-time insights, error detection, and adaptive control mechanisms [23]. Postoperative protocols must include rigorous data collection and outcome tracking to refine algorithms and guide future improvements.

*Decision-making frameworks:* Physicians must balance AI recommendations with clinical judgment, ensuring that human oversight guides all critical decisions [11]. Shared decision-making with patients is vital, discussing the benefits and limitations of AI-assisted surgery transparently. Ethical considerations and patient preferences should shape individualized treatment plans, fostering trust and acceptance in the evolving landscape. By adhering to these criteria and frameworks, healthcare providers can harness the full potential of robotic and AI-assisted surgery delivering safer, more personalized care while navigating the complexities of modern surgical innovation.

## Economic considerations

While clinical benefits are profound, economic considerations often guide healthcare adoption decisions. The literature suggests that despite high initial investments, AI-assisted robotic surgery offers cost-effectiveness through reductions in complications, length of hospital stay, and readmissions. Lai et al. [16] provide an in-depth economic evaluation showing that robotic-assisted surgery, when applied appropriately, leads to significant savings. For example, reduced hospital stays save an average of $1,500 per patient, while fewer postoperative complications translate to savings of $2,000 to $3,000 per surgery. They note that these savings can offset the higher upfront costs over time. Javaid et al. [14] underscore that economies of scale are achieved as surgical teams gain experience, leading to shorter operative times and optimized resource utilization, which further enhances cost-efficiency.

Iftikhar et al. [13] estimate that AI-driven robotic surgeries can reduce reoperation rates by up to 15%, significantly cutting long-term healthcare expenditures. They also emphasize AI’s role in predictive maintenance of robotic systems, reducing downtime and costly repairs. Wah [24] highlights that innovation in AI robotics leads to risk reduction, which in turn lowers malpractice claims and insurance costs, an often overlooked but substantial economic benefit. Conversely, Banbhrani et al. [5] caution that without proper training and integration, costs may increase due to extended operative times and technology underutilization. Hence, investment in surgeon education and infrastructure is critical to realize economic gains.

Moreover, Hölgyesi et al. [12] report that social acceptance of AI-robotic surgery influences utilization rates, which impacts overall economic viability. They stress the need for patient education to enhance confidence and uptake, which would drive cost-effectiveness at scale. Liu et al. [17] review the evolution of surgical robot systems and highlight that as AI algorithms mature and hardware becomes more affordable, the cost–benefit balance will increasingly favor robotic surgery. Thakre and Patel [22] report that in implant dentistry, robotic AI assistance shortens procedure time by 30%, increasing patient throughput and clinic profitability. Xiao et al. [25] demonstrate cost savings from reduced revision surgeries and shorter hospitalizations in spinal surgeries using robotic AI assistance, emphasizing tangible economic benefits alongside clinical gains.

## Economic analysis

The integration of robotics and artificial intelligence (AI) into surgical practice represents a transformative leap in healthcare delivery, promising enhanced precision, reduced complication rates, and improved patient outcomes [1, 24]. However, the economic implications across varied healthcare settings warrant a careful and nuanced examination.

*Cost-effectiveness synthesis:* Robotic and AI-assisted surgeries typically involve significant upfront capital expenditure, including acquisition costs, training, and maintenance of sophisticated systems [4, 16]. Despite these high initial costs, multiple studies have demonstrated favorable cost-effectiveness profiles in medium- to high-volume surgical centers where procedure standardization and efficiency gains maximize technology utilization [10, 15]. AI-driven enhancements, such as intraoperative video analysis and real-time decision support, contribute to reducing operative times and postoperative complications, ultimately lowering overall treatment costs [8, 13]. Conversely, in lower-volume or resource-constrained settings, the fixed costs may outweigh immediate benefits, making widespread adoption financially challenging without subsidization or tailored implementation strategies [12, 16]. The cost-effectiveness improves notably when considering the long-term benefits of reduced hospital stays, lower readmission rates, and improved surgical precision leading to better oncologic and functional outcomes [6, 25].

*Budget impact considerations:* Healthcare systems must balance the financial burden of robotic platforms with their clinical advantages. High-income urban hospitals may absorb these costs through increased surgical volumes and integration with other AI-enhanced diagnostic and therapeutic modalities [5, 21]. Public health payers and hospital administrators should anticipate budget impacts associated with initial procurement, periodic upgrades, and ongoing technical support [16]. In publicly funded or rural healthcare settings, phased implementation approaches, supported by government incentives or public–private partnerships, may mitigate financial pressures [2, 14]. Additionally, digital twin technology and AI-assisted preoperative planning may reduce intraoperative resource use, providing indirect budgetary relief [3, 9].

*Return-on-investment timelines:* ROI timelines for robotic and AI-assisted surgery vary considerably by healthcare setting and surgical specialty. High-volume tertiary centers often observe positive ROI within 3–5 years, driven by procedural efficiency, higher case throughput, and better clinical outcomes translating to lower complication management costs [18, 19]. In contrast, smaller community hospitals may experience longer ROI periods, potentially extending beyond 7 years, depending on case volumes and reimbursement policies [22]. The introduction of AI innovations that optimize workflow and support surgical training can accelerate ROI by expanding the range of procedures amenable to robotic assistance [20, 23].

## Limitations and practical challenges in clinical settings

Despite the technological marvels of AI and robotics in surgery, several limitations hinder their seamless adoption in real-world healthcare systems. One major concern is the high cost of acquisition and maintenance, making these technologies inaccessible for many public and rural healthcare facilities [16, 24]. The complex setup and need for highly trained personnel pose logistical hurdles, especially in resource-constrained regions [10, 13]. Additionally, inconsistencies in surgical outcomes and the limited adaptability of AI algorithms to real-time anatomical variations further dampen clinician confidence [6, 25]. Digital twin technology and real-time image guidance offer promise, but they are still evolving and require high-fidelity data environments to function effectively [3]. Moreover, system interoperability remains a challenge, especially when integrating AI software with diverse robotic platforms and hospital IT systems [4, 15].

## Ethical and legal dilemmas in AI-assisted surgery

The ethical landscape of robotic and AI-assisted surgery is complex and evolving. A central issue is the shifting responsibility in the event of a surgical error should accountability lie with the surgeon, the machine, or the software developers [11]? Ethical frameworks have yet to fully capture these nuances, especially as AI systems begin making semi-autonomous decisions [7, 14]. Concerns over the erosion of surgeon–patient trust are also notable, particularly when decisions are heavily reliant on algorithmic inputs [12]. In pediatric or high-risk surgeries, where human judgment is paramount, the reliance on machine intelligence stirs deeper debates on moral and emotional competencies [5, 10]. Moreover, issues of bias in AI training data often sourced from homogeneous populations raise concerns about equitable treatment outcomes across diverse patient groups [18].

## Regulatory uncertainties and global disparities

The regulatory environment surrounding robotic and AI-driven surgery remains fragmented across regions, with a lack of standardized protocols for validation, approval, and clinical integration [19, 20]. While agencies like the FDA have begun crafting pathways for AI-based medical devices, the rapid pace of innovation often outpaces regulatory reform, leaving many technologies in a gray zone [7, 17]. Inconsistent legal frameworks for data privacy, patient consent, and cross-border data sharing add further complexity [9, 23]. Developing countries face disproportionate challenges due to weaker regulatory oversight and insufficient healthcare infrastructure to support advanced surgical systems [1, 2]. As AI and robotics become more embedded in healthcare, international collaboration will be vital to creating cohesive legal standards that prioritize both innovation and patient safety.

## Discussion and conclusion

The convergence of artificial intelligence and robotics in modern surgery is not just an innovation, it represents a pivotal evolution in how surgical care is conceived and delivered. Across the reviewed literature, a strong consensus emerges: AI-assisted robotic surgery markedly improves surgical precision, patient safety, and clinical outcomes [8, 10, 25]. High-quality studies illustrate how real-time feedback, intraoperative video analysis, and adaptive neuro-visual systems help reduce intraoperative errors, surgical trauma, and postoperative complications [1, 11, 23]. Importantly, these technologies also enhance surgeon performance by reducing fatigue and providing critical decision support, a benefit with ethical implications that reinforce the patient-centered nature of the innovation [11]. The emerging use of digital twin’s virtual patient replicas further supports safety by allowing surgeons to simulate scenarios and anticipate complications [3]. These advances paint a compelling picture of a safer, more informed surgical future.

Equally compelling is the growing body of evidence demonstrating the clinical effectiveness of AI-enhanced robotic procedures across diverse specialties. From urologic oncology to spinal surgery and pediatric cases, AI integration has been shown to reduce operation times, improve surgical margins, and accelerate functional recovery [6, 10, 25]. Technologies, such as predictive analytics, real-time tissue modeling, and vision-enhanced robotic arms, allow for finer dissections and more tailored interventions, directly translating into fewer complications and faster patient recoveries [15, 18, 19]. Even in complex surgeries, AI-assisted techniques prove comparable or superior to conventional methods [13, 21]. The expansion of these tools into fields like dentistry [20] and rehabilitation [2] suggests a scalable model of care, where automation and human expertise converge for better, faster, and less-invasive healing.

However, transitioning these advancements into everyday clinical practice is not without its challenges. While the long-term economic benefits are promising with significant reductions in complications, reoperations, and hospital stays the initial investment in equipment, training, and integration remains substantial [13, 16, 24]. The success of implementation depends heavily on appropriate patient selection, robust training programs, and thoughtful clinical protocols that uphold human oversight alongside AI support [5, 11, 15]. Public acceptance also plays a critical role, as greater awareness and confidence can drive utilization and economic scalability [12]. Encouragingly, as the technology matures and costs decline, the gap between innovation and accessibility is expected to close, paving the way for a more precise, efficient, and equitable surgical future [17, 22].

## Recommendations

To fully realize the potential of robotics and AI in surgery, strategic implementation is essential. Abbasi and Hussain [1] recommend investing in training and infrastructure to equip healthcare professionals with the necessary skills. Ansari et al. [2] advocate integrating AI into existing surgical workflows to enhance decision-making and reduce variability. Asciak et al. [3] emphasize the importance of multidisciplinary collaboration among engineers, data scientists, and surgeons to refine technologies like digital twins. Banbhrani et al. [5] and Balakrishna et al. [4] call for incorporating AI into diagnostics and drug development pipelines to create a cohesive precision medicine ecosystem. Chopra and Ahmed [7] further recommend the development of AI tools that support real-time decision-making during surgeries. Additionally, Wah [24] urges stakeholders to embrace these advancements while maintaining a focus on safety, ethics, and inclusiveness.

## Implications

The integration of AI and robotics in surgery carries wide-reaching implications. Clinically, it enhances surgical precision and patient safety, as evidenced by Bellos et al. [6] and Esposito et al. [10]. Economically, Lai et al. [16] highlight the potential for cost savings through reduced complications and shorter hospital stays. Technologically, innovations like vision-based systems and digital twins [9, 17] open new frontiers for intraoperative navigation and personalized care. Ethically, Haltaufderheide et al. [11] and Hölgyesi et al. [12] stress the need for transparent governance, equitable access, and robust privacy safeguards. From a societal perspective, public trust must be maintained through education, inclusivity, and ethical use of AI.

## Limitations

Despite its promise, AI-robotic surgery faces limitations. High costs and resource demands limit widespread adoption, particularly in low-resource settings [16]. Technical challenges persist, such as algorithmic biases and reliability issues in real-time environments [8]. Additionally, ethical and regulatory uncertainties such as unclear accountability and lack of global standards impede progress [11]. Shahi et al. [21] also point to the need for continuous validation to ensure system safety and surgeon confidence. Furthermore, limited long-term data on AI-robotic surgical outcomes hinder robust comparative analyses [18, 24].

## Future research directions

Future research should focus on enhancing AI model accuracy, reliability, and transparency in real-time surgical settings [7, 17]. Longitudinal studies are needed to evaluate long-term patient outcomes and cost-effectiveness [6, 16]. Research should also explore adaptive and semi-autonomous robotic systems, particularly those powered by large vision models [18]. Studies on digital twins and XR integration for surgical training and simulation [3, 9] will improve preoperative planning. Ethics-focused research must address patient consent, AI accountability, and equitable access [11]. Finally, greater focus should be placed on expanding AI-robotic applications in diverse surgical fields, including pediatrics, dentistry, and spinal surgery [10, 19, 25].

## Conclusion

The fusion of robotics and artificial intelligence is reshaping the future of surgery, bringing remarkable improvements in precision, safety, and efficiency across various medical fields. From cancer treatment to pediatric care and dental procedures, AI-assisted robotic systems are empowering surgeons with innovative tools like digital twins and machine learning-driven insights that lead to better patient outcomes. While the road ahead involves tackling high costs, ethical dilemmas, and ensuring equal access, the potential to minimize complications and speed up recovery makes robotic surgery a vital part of tomorrow’s healthcare. Moving forward, a collaborative effort among clinicians, researchers, and policymakers guided by strong clinical evidence and ethical oversight will be essential to unlock the full promise of the technology and deliver its life-changing benefits to patients everywhere.

## Data Availability

No datasets were generated or analysed during the current study.

## References

[1] Abbasi N, Hussain HK (2024) Integration of artificial intelligence and smart technology: AI-driven robotics in surgery: precision and efficiency. J Artif Intell General Sci 5(1):381–390

[2] Ansari ZJ, Aher A, Thitame SN (2025) Advancements in robotics and AI transforming surgery and rehabilitation. J Pharmacy Bioallied Sci 17(Suppl 1):S46–S48 10.4103/jpbs.jpbs_1937_24 PMC12156781 40511147

[3] Asciak L, Kyeremeh J, Luo X, Kazakidi A, Connolly P, Picard F, Shu W (2025) Digital twin-assisted surgery: concept, opportunities, and challenges. NPJ Digital Med 8(1):32 10.1038/s41746-024-01413-0 PMC11736137 39815013

[4] Balakrishna K, Kumar P, Ridoy MSH, Gadipally V, Banu SB, Dhanraj JA (2024) Robotics and AI integration for automated surgical procedures. In: 2024 international conference on advances in computing, communication and applied informatics (ACCAI). IEEE, pp 1–5

[5] Banbhrani SK, Akhter MN, Noureen F, Talpur MSH (2025) How AI is revolutionizing healthcare: from personalized medicine and diagnostic tools to drug discovery and robot-assisted surgery. Social Sci Rev Archives 3(1):2693–2709

[6] Bellos T, Manolitsis I, Katsimperis S, Juliebø-Jones P, Feretzakis G, Mitsogiannis I, Tzelves L (2024) Artificial intelligence in urologic robotic oncologic surgery: a narrative review. Cancers 16(9):1775. 10.3390/cancers16091775 10.3390/cancers16091775 PMC11083167 38730727

[7] Chopra G, Ahmed S (2025) Artificial intelligence and machine learning–assisted robotic surgery: current trends and future scope. In: Artificial intelligence in biomedical and modern healthcare informatics. Academic Press, pp 23–29

[8] Cizmic A, Mitra AT, Preukschas AA, Kemper M, Melling NT, Mann O, Nickel F (2025) Artificial intelligence for intraoperative video analysis in robotic-assisted esophagectomy. Surgical Endoscopy: 1–10 10.1007/s00464-025-11685-6 PMC12041040 40164839

[9] De Backer P, Matthys R, Rashidian N (2025) AI and XR in robotics. In: Textbook of robotic liver surgery. Springer Nature Switzerland, pp 297–305

[10] Esposito C, Masieri L, Di Mento C, Cerulo M, Del Conte F, Coppola V, Escolino M (2025) Seven years of pediatric robotic-assisted surgery: Insights from 105 procedures. J Robot Surg 19(1):157 10.1007/s11701-025-02257-w PMC12000270 40232570

[11] Haltaufderheide J, Pfisterer-Heise S, Pieper D, Ranisch R (2025) The ethical landscape of robot-assisted surgery: a systematic review. J Robot Surg 19(1):102 40050538 10.1007/s11701-025-02228-1 PMC11885409

[12] Hölgyesi Á, Zrubka Z, Gulácsi L, Baji P, Haidegger T, Kozlovszky M, Péntek M (2024) Robot-assisted surgery and artificial intelligence-based tumour diagnostics: social preferences with a representative cross-sectional survey. BMC Med Inform Decis Mak 24(1):87 10.1186/s12911-024-02470-x PMC10981282 38553703

[13] Iftikhar M, Saqib M, Zareen M, Mumtaz H (2024) Artificial intelligence: revolutionizing robotic surgery. Annals Med Surgery 86(9):5401–5409 10.1097/MS9.0000000000002426 PMC11374272 39238994

[14] Javaid M, Haleem A, Pratap Singh R, Rab S, Suman R, Kumar L (2025) Utilization of robotics for healthcare: a scoping review. J Industr Integr Manage 10(1):43–65

[15] Knudsen JE, Ghaffar U, Ma R, Hung AJ (2024) Clinical applications of artificial intelligence in robotic surgery. J Robot Surg 18(1):102 38427094 10.1007/s11701-024-01867-0 PMC10907451

[16] Lai TJ, Heggie R, Kamaruzaman HF, Bouttell J, Boyd K (2025) Economic evaluations of robotic-assisted surgery: methods, challenges and opportunities. Appl Health Econ Health Policy 23(1):35–49 39333303 10.1007/s40258-024-00920-1

[17] Liu Y, Wu X, Sang Y, Zhao C, Wang Y, Shi B, Fan Y (2024) Evolution of surgical robot systems enhanced by artificial intelligence: a review. Adv Intell Syst 6(5):2300268. 10.1002/aisy.202300268

[18] Min Z, Lai J, Ren H (2025) Innovating robot-assisted surgery through large vision models. Nature Reviews Electrical Engineering, 1–14

[19] Osman EIA, Ismail MMEM, Mukhtar MAH, Ahmed AUB, Mohamed NAAE, Ibrahim AAA, Ibrahim AA (2025) Artificial intelligence and robotics in minimally invasive and complex surgical procedures: a systematic review. Cureus 17(3) 10.7759/cureus.81339 PMC12034508 40296978

[20] Panahi O, Farrokh S, Amirloo A (2025) Robotics in implant dentistry: current status and future prospects. Scientific Arch Dental Sci 7(9):55–60

[21] Shahi A, Bajaj G, GolharSathawane R, Mendhe D, Dogra A (2024) Integrating robot-assisted surgery and AI for improved healthcare outcomes. In: 2024 ninth international conference on science technology engineering and mathematics (ICONSTEM). IEEE, pp 1–5

[22] Thakre D, Patel J (2024) The advancements and benefits of AI-assisted robotic surgery. In: 2024 2nd DMIHER international conference on artificial intelligence in healthcare, education and industry (IDICAIEI). IEEE, pp 1–5

[23] Urrea C, Garcia-Garcia Y, Kern J, Rodriguez-Guillen R (2025) Neuro-visual adaptive control for precision in robot-assisted surgery. Technologies 13(4):135. 10.3390/technologies13040135

[24] Wah JNK (2025) Revolutionizing surgery: AI and robotics for precision, risk reduction, and innovation. J Robot Surg 19(1):1–15. 10.1007/s11701-024-02205-0 10.1007/s11701-024-02205-0 39776281

[25] Xiao X, Wang X, Meng B, Pan X, Zhao H (2025) Comparison of robotic AI-assisted and manual pedicle screw fixation for treating thoracolumbar fractures: a retrospective controlled trial. Front Bioeng Biotechnol 13:1491775. 10.3389/fbioe.2025.1491775 40256780 10.3389/fbioe.2025.1491775 PMC12006081

